# Corrigendum

**DOI:** 10.1111/jdi.13840

**Published:** 2022-07-01

The authors would like to draw the reader's attention to errors in the following article:

Shunsuke Y, Nobuya I. Regulation of glucagon‐like peptide‐1 sensitivity by gut microbiota dysbiosis. *J Diabetes Investig* 2017; 9: 262–264.

In the reference section, Reference 6 is not listed. Figure 1 is based on reference 6. We have included the reference below.

6. Sandrine PC. Will gut microbiota help design the next generation of GLP‐1‐based therapies for type 2 diabetes? *Cell Metab* 2017; 26: 6–7.

The authors apologize for these errors and any inconvenience they may have caused.

